# Analysis of Temperature-Jump Boundary Conditions on Heat Transfer for Heterogeneous Microfluidic Immunosensors

**DOI:** 10.3390/s21103502

**Published:** 2021-05-18

**Authors:** Fraj Echouchene, Thamraa Al-shahrani, Hafedh Belmabrouk

**Affiliations:** 1 Electronic and Microelectronics Lab, Department of Physics, Faculty of Science of Monastir, University of Monastir, Monastir 5019, Tunisia; frchouchene@yahoo.fr (F.E.); ha.belmabrouk@mu.edu.sa (H.B.); 2 Department of Physics, College of Science, Princess Nourah Bint Abdulrahman University, Riyadh 11671, Saudi Arabia; 3 Department of Physics, College of Science at Zulfi, Majmaah University, Majmaah 11952, Saudi Arabia

**Keywords:** biosensors, electrothermal force, immunoassay, microfluidic, temperature jump

## Abstract

The objective of the current study is to analyze numerically the effect of the temperature-jump boundary condition on heterogeneous microfluidic immunosensors under electrothermal force. A three-dimensional simulation using the finite element method on the binding reaction kinetics of C-reactive protein (CRP) was performed. The kinetic reaction rate was calculated with coupled Laplace, Navier−Stokes, energy, and mass diffusion equations. Two types of reaction surfaces were studied: one in the form of a disc surrounded by two electrodes and the other in the form of a circular ring, one electrode is located inside the ring and the other outside. The numerical results reveal that the performance of a microfluidic biosensor is enhanced by using the second design of the sensing area (circular ring) coupled with the electrothermal force. The improvement factor under the applied ac field 15 V_rms_ was about 1.2 for the first geometry and 3.6 for the second geometry. Furthermore, the effect of temperature jump on heat transfer rise and response time was studied. The effect of two crucial parameters, viz. Knudsen number (Kn) and thermal accommodation coefficient (*σ_T_*) with and without electrothermal effect, were analyzed for the two configurations.

## 1. Introduction

There has been a manifest development of nanofluidic devices during the last decades. The exceptional characteristics of these devices have attracted the increasing interest of researchers in several fields such as engineering, microelectronics, biomechanics, and biomedical applications [1,2,3,4,5]. In the biomedical field, many biochemical processes such as mixing [6] and sensing [7,8] can be integrated into a single chip. Biosensors represent powerful tools used in several applications such as drug discovery, medical diagnostics, and security and defense [9]. The interest of microfluidic biosensors is that they offer several advantages such as lower cost, higher sensitivity, faster response time, and lower sample consumption [10]. Immunosensors represent important analytical tools for monitoring antibody–antigen interactions for the detection of appropriate analytes by coupling the immunochemical reaction to the transducer [11,12,13,14,15,16].

Immunosensors can be divided into three classes depending on the type of transducer used: optical, piezoelectric, and electrochemical. Based on the presence or absence of a separation step there are two types of immunoassays: homogeneous immunoassay, where the interaction between antibodies and antigens is made in solution, and the separation step is not required, or heterogeneous immunoassays, where the interaction is made between antibodies immobilized on a solid membrane and the antigen present at the boundary layer that requires the separation of antibody-bound label from the free label before measuring the signal [17,18]. In heterogeneous immunoassays, the concentration of the antigen–antibody complex at the binding surface has a crucial effect. The principal advantage of the heterogeneous immunoassay is its aptitude to concentrate molecules on a reaction surface for easy detection. The reaction rate and the transport phenomena rate by diffusion and convection occurring in heterogeneous immunosensors are related by the Damköhler number, Da. When Da is higher, mass transport is limited, while association kinetic is lower for low Da values [19]. For the microfluidic biosensors, the ratio of the diffusion velocity of molecules to the reaction surface is comparatively small, which causes the development of a diffusion boundary layer limiting the efficiency of biosensors [20,21]. Several methods ranging from passive to active have been developed in order to improve the phenomenon of transport of the analytes to the transducer such as microfluidic confinement [22] and electrothermal effect [23,24,25,26]. When Da is high, mass transport is limited, while association kinetic is not prevailing for low Da values [19]. The effect of Damköhler number has also been analyzed by Selmi et al. [27].

In our previous studies, many approaches were adopted to enhance the reaction rate. The first approach consists of the insertion of a cylindrical or rectangular obstacle within the microchannel near the reaction surface [28,29,30]. The obstacle provokes the modification of the flow near the reaction surface and brings more analyte to it. This leads to an important enhancement in the detection time. The second approach takes advantage of the modification of flow topology generated by flow confinement [27,31]. Some studies benefit from the flow modification induced by the electrothermal force, which leads to an improvement of the biosensor efficiency [28,32,33]. However, these numerical simulations have been done only for two-dimensional configurations and should be extended to 3D geometry. This extension is one of the objectives intended in the present study.

In a recent study, Selmi and Belmabrouk [34] have analyzed the effect of the fluid slip velocity using the Helmholtz–Smoluchowski relationship on the microfluidic biosensor. They have shown that it has an effect on the chemical reaction kinetics. In another recent study, Echouchene et al. performed a simulation of the slip velocity effect in a microfluidic channel under the ac electrothermal effect [35]. The authors have shown that shear stress is intensified by the increase of the applied voltage and the slip length. The influence of the slip velocity will be ignored in the present study since it will focus mainly on the effect of the new proposed geometry as well as the impact of the temperature jump at the fluid–solid interface.

In summary, in the current study, the investigated configuration is a three-dimensional geometry. The influence of the shape of the detection surface, as well as the electrothermal effect on the microfluidic biosensor performance, are analyzed. The impact of the temperature-jump condition at the fluid–solid interface is also investigated.

## 2. Device Geometry and Mathematical Formulation

### 2.1. Device Geometry

To enhance biosensor performance, several strategies may be adopted. In previous papers, we used the flow confinement [27], the insertion of a circular or rectangular obstacle [28,29,30], the electrothermal force [32,33], or a magnetic force [5] in 2D configurations.

**Figure 1:**
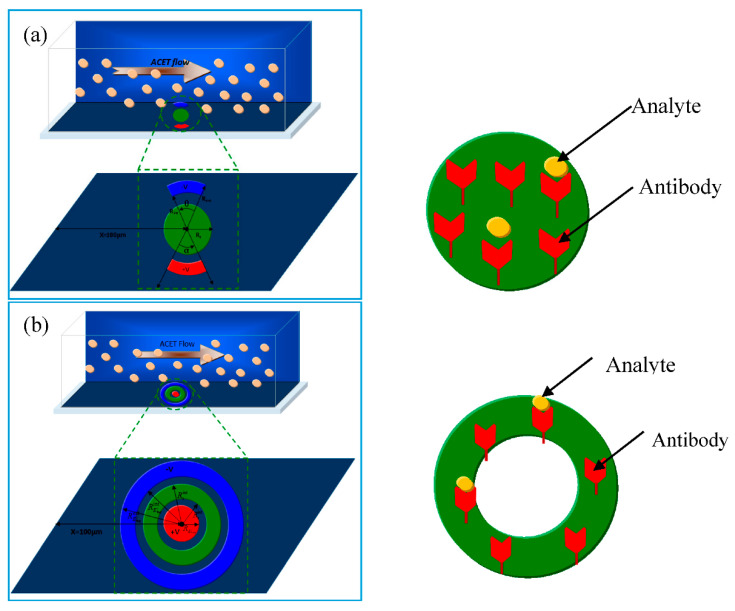
Sketch of two types of 3D models of a microfluidic biosensor. The channel dimensions (length, width, and height) are respectively 250, 50, and 40 µm: (**a**) First configuration: the detection surface is a disk; (**b**) second configuration: the detection surface is a ring.

In the present work, a new geometry of reaction surface and electrodes is proposed. The device is considered to be a 3D parallelepiped (Figure 1). The microchannel used in this study is 50 μm wide, 40 μm high, and 250 μm long. The detection surface and the electrodes are situated in the bottom wall and at a distance X=100 μm from the inlet.

At the device inlet, a small concentration of an analyte is mixed with water. Antibody ligands are fixed on the detection surface. A binding reaction between the antibody and the antigen takes place. The principal aim of this work is to improve the device performance.

Two configurations are proposed. They differ from each other by the shape of the detection surface and the electrodes. Figure 1a presents the first configuration. The detection surface is a disk with a radius $R_{s}=16\;\mu m$. The electrodes are opposite circular crown arches. Their common inner radius is $R_{int}=20\;\mu m$ whereas their external radius is $R_{ext}=25\;\mu m$. The aperture angles of the cathode (positive electrode) and the anode (negative electrode) are respectively denoted by α and θ. These angles are adjustable and several values varying in the range from 40° to 160° will be tested.

Figure 1b presents the second geometry. The detection surface is a circular crown surrounded by the electrodes. Its inner radius is $R_{s,int}=15\;\mu m,$ and its outer radius is $R_{s,ext}=22\;\mu m$. The positive electrode is a disk having a radius $R_{c}=10\;\mu m$. The negative electrode is a circular crown. Its inner radius is $R_{a,int}=25\;\mu m,$ and its outer radius is $R_{a,ext}=30\;\mu m$.

In both models of biosensors, the area of the reaction surface is taken equal to S_R_ = 800 µm^2^.

### 2.2. Transport Equations and Adsorption Model

The flow velocity field in the microchannel is calculated by the continuity and Navier–Stokes equations. In this study, the fluid is assumed to be Newtonian and incompressible. The flow is laminar and steady but is not isotherm. The continuity and motion equations can be written as follows:

(1) $$\{\begin{array}{c} \nabla .\vec{U}=0\\ \rho \vec{U}.\nabla \vec{U}=-\nabla p+\mu \nabla^{2}\vec{U}+{\vec{F}}_{e} \end{array}$$

where $\vec{U}$ is the velocity, ρ is the density, p is the pressure and μ is the dynamic viscosity.

The application of a non-uniform AC electric field, $\vec{E}$, on a fluid provokes a temperature gradient due to the Joule effect. The latter essentially depends on the electrical conductivity of the solution (fluid) and on the amplitude of the electric field. Therefore, local variations of the electrical conductivity, σ, and the permittivity, ε, occur. This leads to an additional force applied to the fluid. This force is given by [28,32,33]:

(2) $${\vec{F}}_{e}=-\frac{1}{2}\left\{\left(\frac{\nabla \sigma }{\sigma }-\frac{\nabla \varepsilon }{\varepsilon }\right).\vec{E}\frac{\varepsilon \vec{E}}{1+\omega^{2}\varepsilon^{2}/\sigma^{2}}+\frac{1}{2}{\left|\vec{E}\right|}^{2}\nabla \varepsilon \right\}$$

where ω is the angular frequency of the AC electric field. This force will have an important role and may modify substantially the flow and, hence, the biosensor features. The fluid investigated is supposed to possess the same properties as water. In the range of temperatures close to 300 K, we have [36] ∇ε/ε=−0.04 ∇T and ∇σ/σ=0.02 ∇T.

The energy equation is given by

(3) $${\rho C}_{p}\vec{U}.\nabla T=k\nabla^{2}T+\sigma {\left|\vec{E}\right|}^{2}$$

where k is the thermal conductivity and $C_{p}$ is specific the heat capacity. In addition to terms due to heat convection and diffusion, the above equation contains a source term due to the Joule effect. The above equations are coupled and should be solved together iteratively.

The electric field is computed by solving Poisson–Laplace equation.

The equation of the transport of the antigen in the bulk liquid phase is given by

(4) $$\frac{\partial C}{\partial t}+\vec{U}.\nabla C=D\nabla^{2}C$$

*C* is the bulk concentration (mol/m^3^), D is the diffusion coefficient of the antigen, and t is the time. This equation contains a cumulative transient term, a convection term, and a diffusion term. However, no source term is involved. Indeed, the binding reaction takes place only on the sensitive membrane that is located in a boundary of the microchannel.

**Figure 2:**
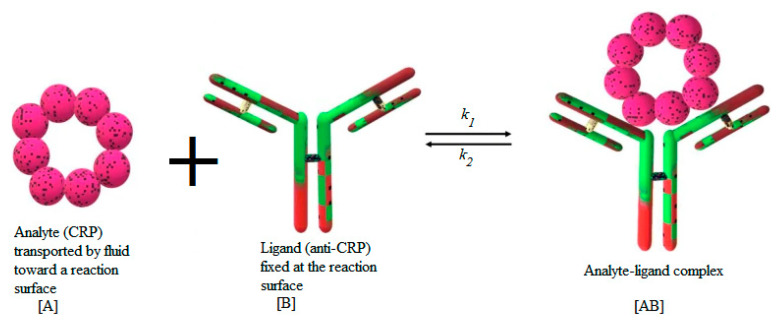
Schematic illustration of C-reactive protein (CRP) binding reaction.

Many models are available in the literature to represent the adsorption of the analyte A. This process may be monolayer or multilayer. It may be due to physical forces or chemical covalent binding. The models may be empirical or based on statistical physics [37,38]. In this study, the first-order Langmuir model is employed to characterize the adsorption reaction between the antibody B fixed at the reaction surface and the suspended target analyte A. Figure 2 illustrates the process of the binding reaction between the analyte and the ligand.

The formed complex is designated by AB. In this study, the CRP protein is considered as an analyte for the analysis of the binding kinetics with FITC-conjugated monoclonal sheep anti-human C-reactive protein (CRP) antibody. The anti-CRP (ligand) is immobilized onto a plain silicon nitride waveguide with diffraction grating. After flow cell attachment, CRP (analyte) is introduced and binding monitored by fluorescence-based evanescent field detection [22].

The binding force may have physical or chemical origins. The activation energy may vary in a wide range. The rate of adsorption and desorption involved in the above equation are denoted by $k_{1}$ and $k_{2}$, respectively. The number of active sites available in the sensitive membrane is an important parameter. In addition, in microfluidic applications, the fluid topology may also play an important role to enhance the adsorption reaction. This property is exploited in the present study.

For the adopted Langmuir model, the concentration [AB] of the antigen–antibody complex is given by

(5) $$\frac{\partial \left[AB\right]}{\partial t}=k_{1}{\left[A\right]}_{surf}\left(\left[B_{0}\right]-\left[AB\right]\right)-k_{2}\left[AB\right]$$

where ${\left[A\right]}_{surf}$ denotes the analyte concentration on the surface and $\left[B_{0}\right]$ represents the concentration of free antibodies. It is related to the total number of active sites accessible on the sensor surface. Obviously, the complex AB is confined on the sensor surface.

### 2.3. Boundary Conditions

For the dynamic field, the following boundary conditions are adopted.

- At the inlet, a parabolic profile is adopted.
- At the channel exit, a fully developed flow condition is adopted.
- At lateral walls, a no-slip condition is adopted.

In a previous publication, the influence of the slip velocity has been investigated in an AC electrothermal micropump [35]. However, in the present work, the impact of the slip velocity will be ignored.

In the matter of the energy equation, the conduction heat flux is zero at the inlet and outlet boundaries. An insulation condition is applied to the lateral walls (except the electrodes). The electrodes are maintained at a constant temperature or a temperature-jump condition is applied as will be explained hereafter.

Concerning the electrical field, all the walls except the electrodes are assumed to be electrically insulated. However, an AC voltage is applied to the electrodes. The rms (root mean square) voltages are $\pm V_{rms}$.

Concerning the analyte transport equation, the concentration, $C_{0}$, of the analyte at the inlet is maintained constant. At the outlet, the diffusion is equal to zero. The lateral walls except the reaction surface are impermeable. No reaction occurs between these walls and the analyte. Finally, at the reaction surface, an equilibrium occurs between the diffusion flux on one hand and adsorption and desorption rates on the other hand. This boundary condition links Equations (4) and (5).

### 2.4. Numerical Method and Algorithm

The numerical method used to discretize the above equations and perform the simulations is the finite element method. The application of the well-known Galerkin method enables to transform the governing equations into a linear or nonlinear matrix form:

(6) $$KX=Q\;\;\;\mathrm{or}\;\;\;H\frac{dX}{dt}+KX=Q$$

where K is the stiffness matrix, X and Q are the unknown and load vectors, and H is the damping matrix. Poisson–Laplace, Navier–Stokes, and energy equations are time-independent. To obtain the solution X, only a matrix inversion algorithm is required. However, the antigen transport equation and the Langmuir model should be solved in the transient regime. Therefore, a scheme to discretize the time derivative should be used.

The computation domain is split into an unstructured mesh containing triangular elements. Particular care is paid to the areas of the binding surface and the electrodes. Indeed, mesh refinement is undertaken to ensure a satisfactory convergence of the solution.

The algorithm followed to solve the governing is as follows:

- Solve the Poisson–Laplace equation to obtain the voltage and the electrical field, $\vec{E}$.
- Simultaneously solve Navier–Stokes and energy equations to deduce the dynamic and thermal fields, i.e., $\vec{U}$ and T.
- Solve the antigen transport equation and the Langmuir model to obtain the temporal evolution of the concentrations, C and [AB]. These equations are time dependent.

Table 1 summarizes the values of the physicochemical parameters required for the computation of the different quantities. They are similar to those available in the literature [21,39,40]. The kinetic parameters of monoclonal native pentameric pCRP/Anti-CRP given by [40] are used in this work.

## 3. Results

### 3.1. Model Validation

The chemical binding kinetics of the analyte (anti-rabbit IgG) with the ligand (rabbit IgG) at the reaction surface has been validated with experimental results of Hofmann et al. [22] without confinement flow and ac applied voltage. For the immunoassay application, rabbit IgG is immobilized to a circular detection area 4 mm in diameter and Cy-5-labeled anti-rabbit IgG is introduced as analyte in the sample flow. The analyte solution was 10 nM anti-rabbit Cy5 IgG in 1% *w*/*v* Bovine Serum Albumin- phosphate-buffered saline (BSA-PBS) while ∼3 pmol rabbit IgG in OptoDex matrix is immobilized on the sensitive surface [22]. The association rate constant, *k*_1_, and dissociation rate constant, *k*_2_, for IgG-anti-IgG binding interactions are 2.5 × 10^5^ M^−1^/s and 3 × 10^−4^ s^−1^, respectively [21]. The diffusion coefficient of IgG is 3 × 10^−11^ m^2^/s [21,22].

**Figure 3:**
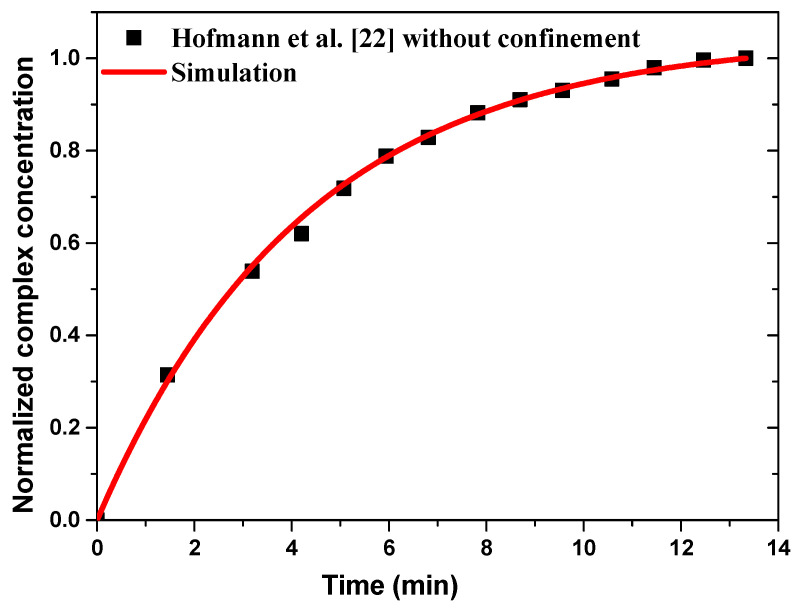
Temporal evolution of the normalized average surface concentration of the complex compared to the experimental data reported by Hofmann et al. [22].

Figure 3 illustrates the numerical results of normalized complex concentration as a function of time compared to the experimental data without confinement and for a sample flow rate fixed at 66 µL/min. A good agreement is observed between the present results and the experimental data of Hofmann et al. [22].

In order to optimize microfluidic biosensor performance, the effects of ac applied voltage and thermal boundary conditions for two biosensor geometries were studied. The numerical calculation is carried out in 3D.

### 3.2. Effect of Surface Reaction Shape

**Figure 4:**
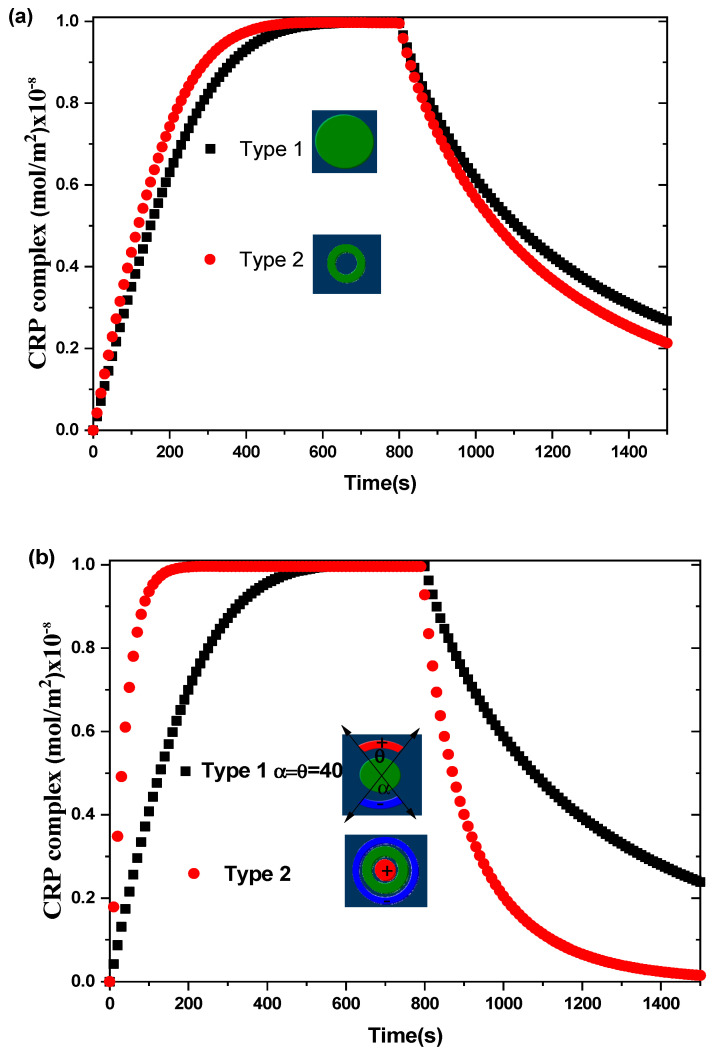
Transient evolution of the average surface concentration of CRP complex for the two types of biosensors for: (**a**) V_rms_ = 0 V and (**b**) V_rms_ = 15 V.

Figure 4 shows the transient evolution of the CRP complex for the two biosensors proposed. The simulations are performed without and with an alternating applied voltage at V_rms_ = 15 V and for an average inlet velocity u_ave_ = 100 µm/s. The aperture angles, θ and α, characterizing the electrodes for the first biosensor model are taken to be equal to 40°. The other physical and geometric parameters used here are listed in Table 1. The curves exhibit the average surface concentration of CRP-anti-CRP complexes ${\left[AB\right]}_{surf}$ as a function of time. This quantity is defined as the average spatial value over the reaction surface area $S_{R}$:

(7) $${\left[AB\right]}_{surf}=\frac{1}{S_{R}}\iint_{S_{R}}^{}\left[AB\right]\left(x,y,z=\right)dxdy$$

In the case of V_rms_ = 0 V (Figure 4a), the binding reaction rate related to the circular reacting surface without electrothermal force is smaller than that related to the reaction surface having the form of a circular ring.

In the case of V_rms_ = 15 V (Figure 4b), a significant improvement in the binding reaction is observed for the second model compared to the first model. The electrothermal force generated by the electrodes for the second model contributes effectively to the increase of the reaction rate. The enhancement factor defined as the ratio of the slope of binding reaction with ac applied voltage to that without ac applied voltage is about 1.2 for the first model and 3.6 for the second model of the proposed biosensors. Therefore, the shapes of the reaction surface and the electrode arrangement have an important role in improving the biosensor response.

In order to show the efficiency of our designs, we compare our results with those found by Huang et al. [40] in terms of initial slope (see Table 2) with and without applied voltage.

The results illustrated in the table show a considerable improvement in the chemical binding kinetics using the second proposed biosensor model. The results achieved by our second model without applied voltage is almost the same as that found by Huang et al. [40] under V_rms_ = 15 V, keeping the same reaction surface area.

### 3.3. Effect of Thermal Boundary Conditions

The study of fluid flow and heat transfer in microsystems requires taking into account the effect of rarefaction, which appears when the number of Knudsen, which is the ratio of the mean free path to the hydraulic diameter, is between 10^−3^ and 10^−1^. The first-order temperature-jump boundary condition [41,42,43,44,45] is widely used to solve the heat equation:

(8) $$T-T_{w}=\frac{2-\sigma_{T}}{\sigma_{T}}\frac{2\gamma }{\gamma +1}\frac{1}{Pr}\Lambda \nabla_{n}T$$

where *T_w_* is the wall temperature, $\sigma_{T}$ is the energy accommodation coefficient, *γ* is the ratio of the specific heat capacities, $Pr={\mu C}_{P}/k$ is the Prandtl number, Λ is the mean free path, and $\nabla_{n}T$ is the temperature gradient normal to the surface.

In this subsection, the effect of the temperature-jump boundary condition at the wall is considered in the simulation and compared with the results obtained without temperature jump. Two cases are therefore considered. In the first case, no jump temperature is applied. Therefore, the electrodes are maintained at a fixed temperature T = T_0_ = 300 K, whereas the other walls of the micro-channel are insulated. In the second case, the temperature-jump condition is applied to the electrodes only. However, the other walls are insulated.

#### 3.3.1. Effect of Jump Temperature on Temperature Rise

**Figure 5:**
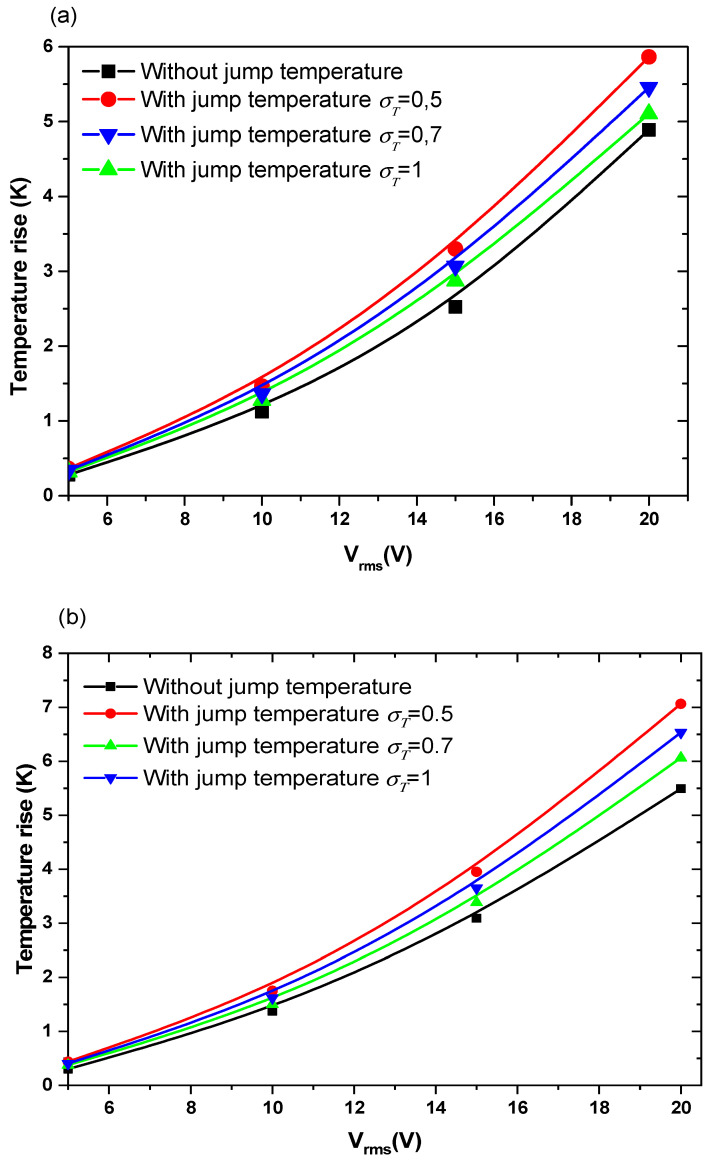
Temperature rise versus the applied voltage for first structure and for Knudsen number (Kn) = 0.02 and: (**a**) θ = 40°, (**b**) θ = 160°.

**Figure 6:**
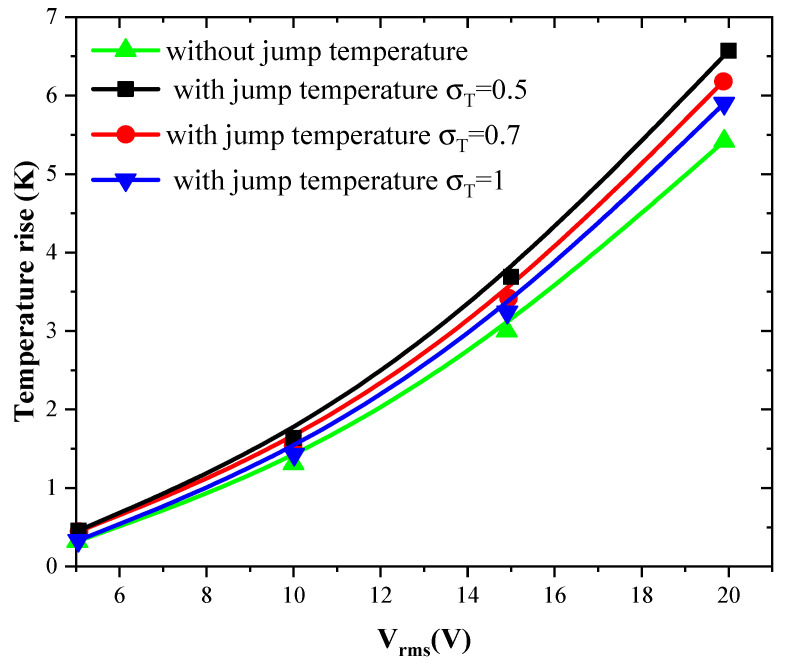
Temperature rise versus the applied voltage for second structure and for Kn = 0.02.

Figure 5 and Figure 6 show the temperature rise as a function of applied voltage with and without temperature-jump boundary condition for both structures for Knudsen number Kn = 0.02, u_ave_ = 100 µm/s, and *f* = 100 kHz. Knudsen number, Kn, is the ratio of the molecular mean free path, Λ, to the characteristic geometric high of microchannel H. Figure 5a,b is related to the first geometry with electrode aperture angles α=θ=40° and α=θ=160°. Figure 6 is related to the second geometry.

Three values of thermal accommodation coefficient, $\sigma_{T}$ = 0.5, 0.7, and 1 are tested. This coefficient represents the fraction of molecules which, after striking the wall, acquire an average total energy identical to that of the molecules of the fluid at the wall temperature T_w_. In this study, the wall temperature is taken equal to 300 K.

The variation of the temperature due to the new boundary condition depends on the geometry of the detection surface and the electrode shape. It depends also on the applied voltage and the thermal accommodation coefficient, $\sigma_{T}$. When the applied voltage, the temperature of the fluid increase with an amount of about 3 to 4 K when V_rms_ = 15 V. This increase versus V_rms_ has a parabolic trend.

The quantity $\left(2-\sigma_{T}\right)/\sigma_{T}$ present in Equation (8) takes the values 3, 1.9, and 1 when the thermal accommodation coefficient is respectively equal to 0.5, 0.7, and 1. It is clear that the temperature rise increases when the $\sigma_{T}$ decreases. In other words, the boundary condition will have an effect on the biosensor answer and should be taken into account to simulate accurately the detection process. The influence of the temperature-jump condition, through the thermal accommodation coefficient, is accentuated for high applied voltages.

In order to take into account the effect of the temperature-jump condition, our calculation results were compared with the results from Huang et al. [40] in which the slip condition was not taken into account.

It should be noted from the results of Table 3 that the energy exchange that occurs between a molecule and a solid surface struck by this molecule, given by the accommodation coefficient, is an essential and important parameter during the design of nanofluidic biosensors.

The above comments are valid for the two configurations and for any value of the aperture angles, α and θ. Only the amplitude of the temperature rise varies slightly from one configuration to another. The boundary conditions have an impact on the temperature and consequently on the electrothermal force. This provokes a modification of the velocity field near the detection surface.

**Figure 7:**
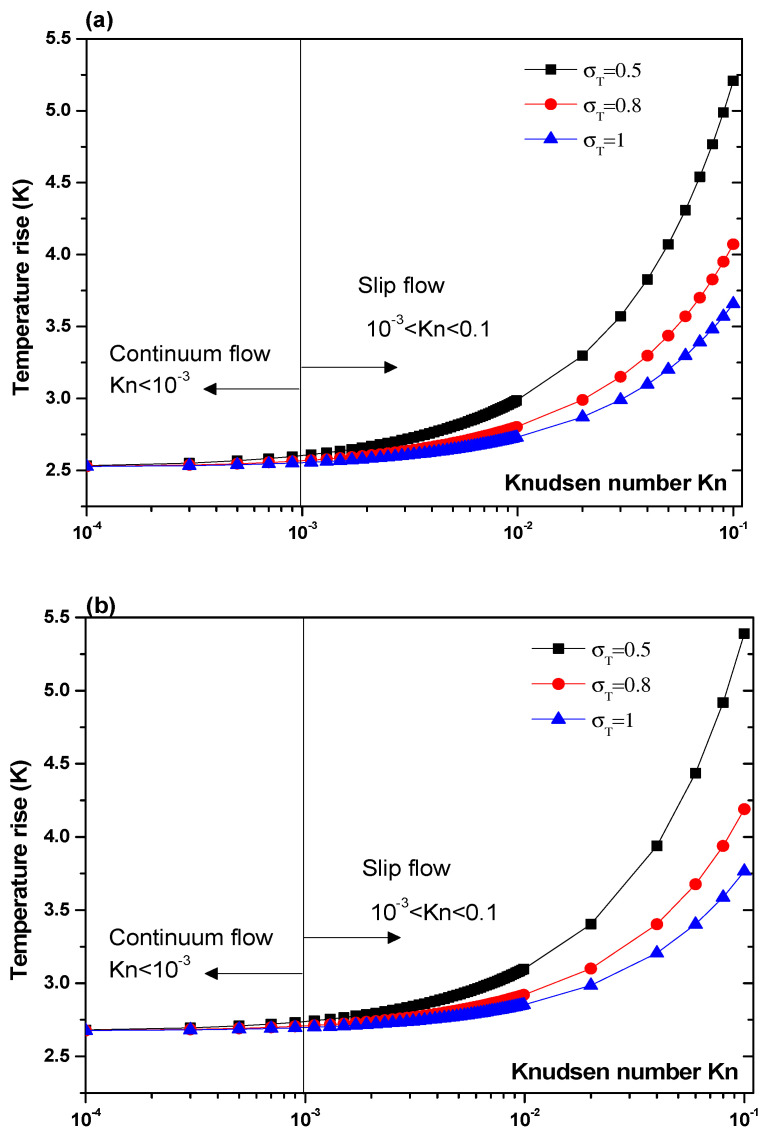
Temperature rise versus the Knudsen number for first structure and for V_rms_ = 15 V, and: (**a**) θ = 40°, (**b**) θ = 160°.

**Figure 8:**
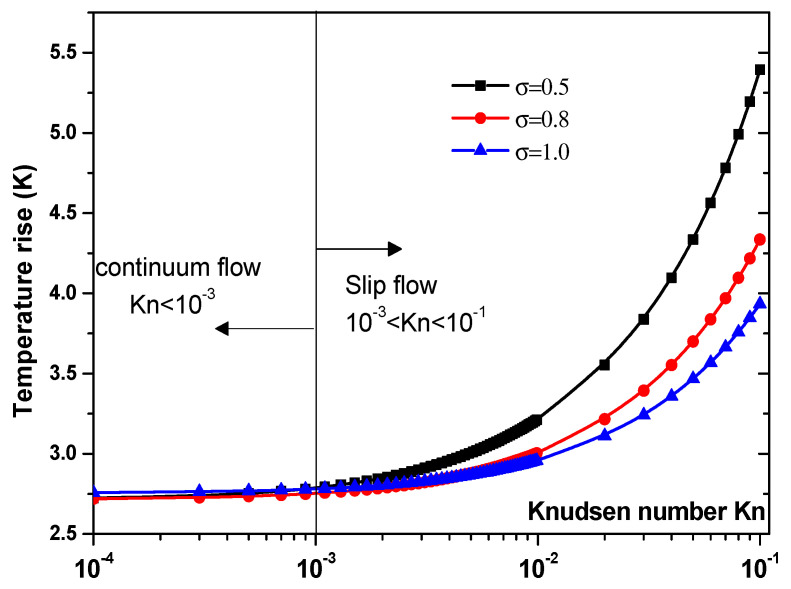
Temperature rise versus the Knudsen number for second structure at V_rms_ = 15 V.

Another parameter that is expected to impact the biosensor characteristics is the Knudsen number, Kn. The effect of this number should be elucidated. Figure 7 and Figure 8 illustrate the temperature rise versus the Knudsen number, Kn, for three values of the thermal accommodation coefficient, $\sigma_{T}$ = 0.5, 0.8, and 1. They correspond respectively to the first geometry and the second geometry. Regarding the first geometry, two cases are considered. Indeed, the electrode aperture angles are α=θ=40° for Figure 7a and α=θ=160° for Figure 7b. According to these figures, two regimes can be observed based on the value of Kn. They are the slip flow (10^−3^ < Kn < 0.1) and continuum flow regimes. This numerical result is in agreement with that reported by Yang et al. [45], who took into account the effects of both temperature jump and slip velocity close to the wall. The temperature jump on temperature rise has an insignificant effect, in the continuum flow regime, and especially for $\sigma_{T}$ = 1.

In the slip flow regime, the effect of temperature-jump condition on the temperature rise becomes more important, particularly for σ_T_ = 0.5. We can conclude from these results that taking into account the temperature jump is of capital importance for the analysis of the heat transfer of nanofluids biosensors.

#### 3.3.2. Effect of Jump Temperature on Response Time

The reaction surface shape, the temperature jump, and the Knudsen number also have an impact on the response time, $T_{R}$, of the biosensor. Many relations may be adopted to define this parameter. Indeed, the response time may correspond to the time for which the complex concentration, [AB], reaches its maximal value. This quantity is directly related to the slope of the CRP complex concentration curves.

**Figure 9:**
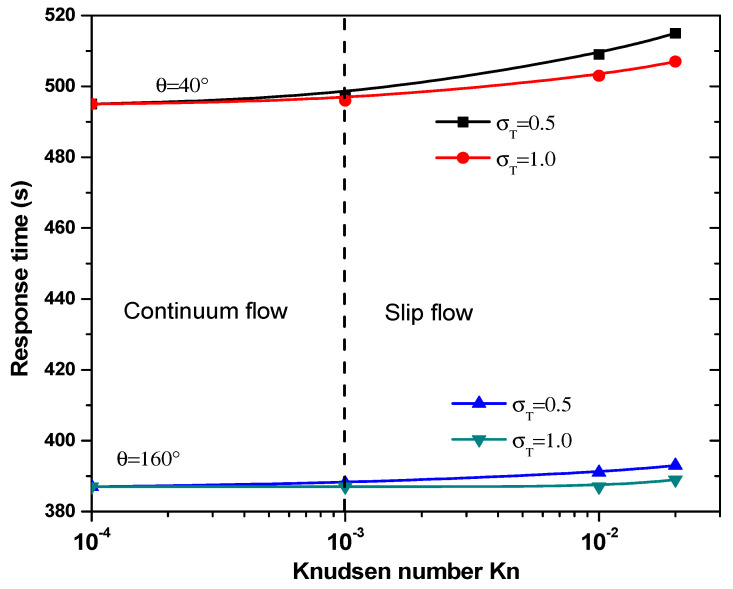
Response time versus the Knudsen number for first structure.

The equilibrium binding time, *T_R_*, as a function of the Knudsen number for θ = 40° and 160° is plotted in Figure 9 for two values of thermal accommodation coefficient, $\sigma_{T}$. It is clear that the temperature jump has minor effect on response time, *T_R_*, in the continuum flow regime. In slip flow regime, response time increases significantly especially for $\sigma_{T}$ = 0.5. The main factor controlling the response time, $T_{R}$, is obviously the electrode aperture angles. Indeed, the effect of the number $\sigma_{T}$ is dominated by the electrode shape.

**Figure 10:**
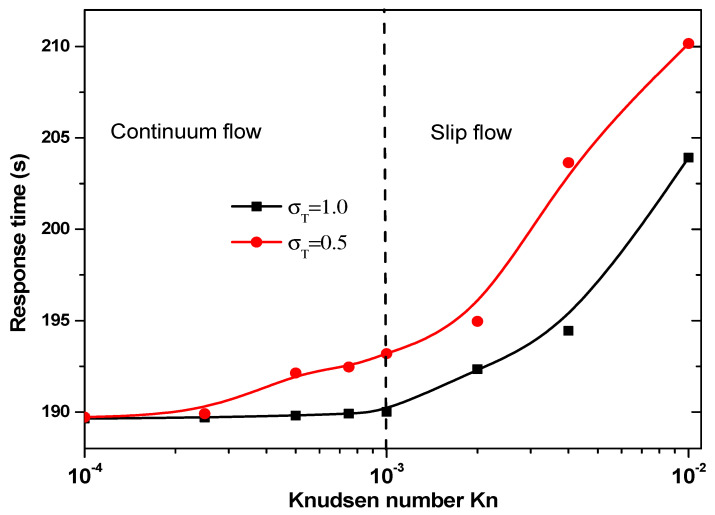
Response time versus the Knudsen number for second structure.

Figure 10 presents the response time of biosensor versus the Knudsen number for $\sigma_{T}$ = 0.5 and 1 for the second geometry. Two regimes can be observed based on the value of Kn. The thermal accommodation coefficient affects the start of slip flow. For $\sigma_{T}$ = 1, the slip regime is started for Kn > 10^−3^, then for $\sigma_{T}$ = 0.5 this regime is started for Kn > 5 × 10^−4^.

The answer time of the second geometry caries in the range 190 to 210 s according to Knudsen number and the thermal accommodation coefficient, $\sigma_{T}$. It is significantly smaller than that related to the first geometry.

## 4. Conclusions

A 3D numerical analysis was carried out to study the immunoassay in a biosensor for two forms of reaction surface with and without temperature-jump boundary condition. The effect of electrothermal force was analyzed. The effects of several important parameters were discussed, namely, the applied voltage, the reaction surface shape, the thermal accommodation coefficient, and the Knudsen number. From the numerical results found in this work, several conclusions can be drawn:

- The performance of the microfluidic biosensor can be further enhanced by using the second design of the sensing area (circular ring) coupled with the electrothermal force.
- Taking into account the temperature jump in the vicinity of the wall of the microchannel is very important, especially in the slip flow regime (Kn > 10^−3^).
- Neglecting the temperature jump induces to overestimate temperature rise for biomedical applications and response time for microfluidic biosensors.
- The effect of thermal accommodation coefficient appears in slip flow.

In conclusion, a reaction surface in the form of a circular ring improves the performance of the biosensor. In addition, it is primordial to take into account the temperature jump especially in the slip flow regime for medical application.

## Figures and Tables

**Table 1:** Numerical values of the parameters used in the current simulations [21,39,40].

Parameter	Unit	Value
$k_{1}$	$m^{3}/\left(\mathrm{mol}.s\right)$	$10^{4}$
$k_{2}$	$s^{-1}$	$2.6\times 10^{-2}$
$B_{0}$	$\mathrm{mol}/m^{2}$	$1.4\times 10^{-8}$
D	$m^{2}/s$	$2.175\times 10^{-11}$
$C_{0}$	$\mu \mathrm{mol}/m^{3}$	6.4
k	W/(K.m)	0.6
ρ	$\mathrm{kg}/m^{3}$	1000
μ	Pa.s	$1.08\times 10^{-3}$
$C_{p}$	kJ/(kg.K)	4.184
σ	S/m	$5.75\times 10^{-2}$
$\varepsilon_{r}$		80.2

**Table 2:** Comparison of initial slope (×10^−11^) for our proposed models with the results of Huang et al. [40].

	V_rms_ = 0 V	V_rms_ = 15 V
First model (θ = 40°)	3.64	4.39
Second model	4.53	17.4
Huang et al. [40] (Type-4)	1.48	4.51

**Table 3:** Comparison of temperature rise with and without temperature jump with the results of Huang et al. [40].

Applied Voltage (V)		5	10	15	20
Temperature rise (K) [40]		0.31	1.31	2.73	4.93
Temperature rise (K), first model (θ = 160°)	Isothermal	0.301	1.373	3.089	5.492
	Jump temperature $\sigma_{T}$ = 1	0.374	1.499	3.383	6.063
Temperature rise (K) second model	Isothermal	0.28	1.375	3.15	5.65
	Jump temperature $\sigma_{T}$ = 1	0.33	1.42	3.22	5.83

## Data Availability

Not applicable.
